# Emergence of active nematics in chaining bacterial biofilms

**DOI:** 10.1038/s41467-019-10311-z

**Published:** 2019-05-23

**Authors:** Yusuf Ilker Yaman, Esin Demir, Roman Vetter, Askin Kocabas

**Affiliations:** 1 Department of Physics, Koç University, 34450 Sarıyer, Istanbul Turkey; 2 Bio-Medical Sciences and Engineering Program, Koç University, 34450 Sarıyer, Istanbul Turkey; 3 Department of Biosystems Science and Engineering, ETH Zurich, 4058 Basel, Switzerland; 4 Koç University Surface Science and Technology Center, Koç University, 34450 Sarıyer, Istanbul Turkey

**Keywords:** Biological physics, Topological defects

## Abstract

Growing tissue and bacterial colonies are active matter systems where cell divisions and cellular motion generate active stress. Although they operate in the non-equilibrium regime, these biological systems can form large-scale ordered structures. How mechanical instabilities drive the dynamics of active matter systems and form ordered structures are not well understood. Here, we use chaining *Bacillus subtilis*, also known as a biofilm, to study the relation between mechanical instabilities and nematic ordering. We find that bacterial biofilms have intrinsic length scales above which a series of mechanical instabilities occur. Localized stress and friction drive buckling and edge instabilities which further create nematically aligned structures and topological defects. We also observe that topological defects control stress distribution and initiate the formation of sporulation sites by creating three-dimensional structures. In this study we propose an alternative active matter platform to study the essential roles of mechanics in growing biological tissue.

## Abstract (web summary)

Active nematics consist of elongated self-driven units and can emerge in bacterial systems. Here the authors investigate such emergence in a growing bacterial biofilm and demonstrate that localized stress and mechanical instabilities drive the formation of large-scale order.

## Introduction

Biofilm formation is a collective response of bacteria^1–6^. Depending on the availability of food and environmental conditions^7^, *B. subtilis* produces matrix proteins and initiates the formation of a biofilm^2^. During biofilm development, motile bacteria differentiate into an aligned chain of cells. Growing chains further develop fibers and bundles, which shape the overall biofilm morphology^8–13^. These distinct aligned structures promote sliding of a colony on a solid surface, where the swimming behavior is not efficient^14^.

Aligned cellular structures are also observed in a variety of biological phenomena. During wound healing, migrating cells align and form fingering structures at the leading edge of the tissue^15^. Similarly, cultured cells^16–18^ and isolated bacteria colonies can form nematic alignment and modulate the cellular density and active stress^19–23^.

Recent studies have shown that liquid crystal theory can provide a suitable framework to study the dynamics of growing tissue as an active nematic system. Mainly, the dynamics of cellular alignment, topological defects, and edge instabilities have been explored^16–20^. During growth, mechanical instabilities also play essential roles by generating a large structural folding. Growing aligned fibers and bundles can accumulate stress and buckle. The dynamics of active nematics (AN) and mechanical properties of growing material are intricately coupled. However, we do not know how the mechanical instabilities drive the dynamics of active nematics and form ordered structures.

Here, by investigating the formation of a bacterial biofilm starting from a single bacterium, we study the detailed mechanical instabilities driving the formation of a nematic active matter. We reveal the direct relation between local stress, localized buckling, and edge instabilities. This system provides new mechanical insights to explore the complex dynamics of biological AN^24–28^.

## Results

### Formation of chaining bacterial biofilms

**Fig. 1:**
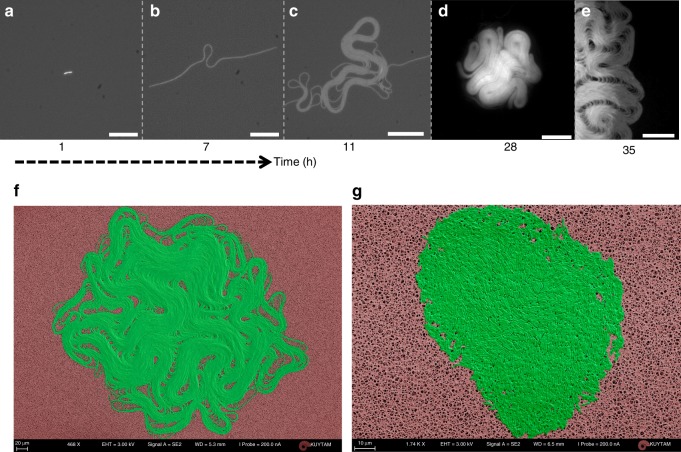
Emergence of active nematics in a bacterial biofilm. Snapshots of a growing biofilm. **a** The bacterium initially elongates and forms a bacterial chain through proliferation (*t* = 2.5 h). Scale bar, 30 μm. **b** Due to local stress, the chain of attached bacteria buckles (*t* = 7 h). Scale bar, 30 μm. **c** Multilayered and circular structures appear at the region where the localized buckling takes place (*t* = 11 h). Scale bar, 30 μm. **d** The biofilm formation (*t* = 28 h). Scale bar, 150 μm. **e** Kink bands appear and separate different domains across the biofilm. Scale bar, 30 μm. Scanning electron microscopy images of **f** the biofilm formed by GFP-labeled laboratory strain 168 (BAK47) and **g** the isolated bacterial colony formed by the non-chaining strain. All colonies were grown at *T* = 21 °C

We first used time-lapse fluorescence microscopy to observe the temporal evolution of a biofilm formation. Figure 1 shows the snapshots from the growth of a chaining *B. subtilis* biofilm, laboratory strain 168 (Fig. 1). This strain apparently forms a biofilm on a solid agar surface. Initially, divided cells give rise to a long and straight chain of attached bacteria. After several cell divisions, the first mechanical instability occurs (Supplementary Movie 1).

Interestingly, unlike Euler instability, this initial buckling is very localized. As the bacterial chain grows, the buckled region forms a crumpled structure (Fig. 1c). Some parts of this structure become multilayered and circular. Similarly, these multilayered structures continue to grow radially, and they split into two (Supplementary Figure 1). Moreover, sharp walls appear across the biofilm, and these bands are observed as dark lines separating different domains in fluorescence images (Fig. 1d, e). All the basic dynamics and shapes described above were observed repeatedly across the biofilm and generate perfectly aligned cellular structures (Fig. 1f). This aligned structure and its dynamics strongly resemble the nematic active matter systems, particularly microtubule-based AN^27,28^ (Supplementary Movies 2–4).

To clarify the structural differences between a nematic biofilm and an isolated bacterial colony, we grew a similar but non-chaining *B. subtilis* strain (BAK51, a derivative of 3610) under the same environmental conditions. This wild isolate failed to form chaining on an agar surface, but can form a biofilm at a later stage stochastically (Supplementary Movie 5). In our bacterial strains, the flagella- producing gene (*hag*) was also mutated to eliminate the swimming-induced motion. Detailed scanning electron microscopy (SEM) images show tightly packed cells with fairly smooth colony edges (Fig. 1g, Supplementary Fig. 2).

Clear nematic alignment of the biofilm required optimization of several parameters. First, we reduced the growth rate by decreasing the ambient temperature to eliminate the twisting and breakage of a bacterial chain (Supplementary Fig. 3). Second, green fluorescent protein (GFP) labeling is essential to observe finely aligned structures, but low-power fluorescence excitation is also necessary to eliminate light toxicity during biofilm development.

### Localized buckling

**Fig. 2:**
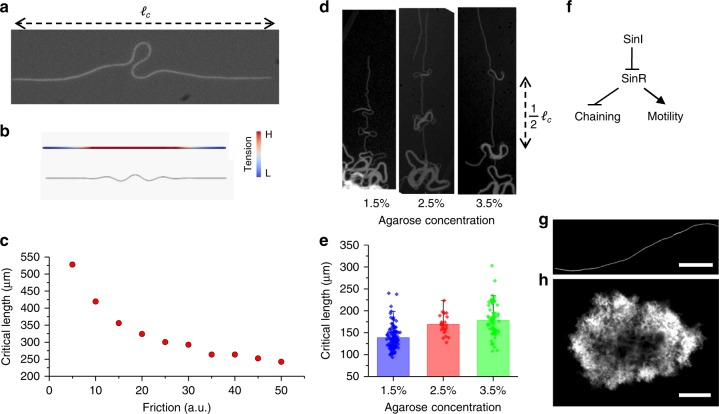
Localized buckling of a bacterial chain. **a** A snapshot of a bacterial chain with a length just above the critical buckling length. The buckled region is highly localized due to **b** localization of stress generated by the uniform friction force. The stress accumulates from the tip to the center of the chain due to outward axial velocity. The uniform friction force creates an almost linear stress distribution along the chain. The snapshots of the simulation before and after the buckling. Color code shows the tension at the pre-buckling stage. **c** Plot of the critical buckling length against friction, simulation results. **d** Successive localized buckling instabilities at 1.5%, 2.5%, and 3.5%, respectively. After the first buckling, the subsequent buckling occurs at the middle of the straight part of the chain, which implies that the stress is symmetric around the center, and the connection of the former buckling region with the straight part is effectively a free end. Therefore, the separation between buckled regions is equal to half of the critical buckling length. **e** Experimental results of the critical buckling length for various agarose concentrations. The data are collected from more than 100 chains. Error bars are defined as s.d. **f** Schematic of a simplified regulatory network that controls the biofilm formation. The protein sinI regulates the chaining state through double-negative feedback. **g, h** Bacterial chain grown in liquid LB. **g** The chaining formation is initiated by IPTG induction. **h** In the later stages, the colony forms a biofilm via supercoiling. Scale bar, 50 and 200 μm

Localized buckling appears to be the first building block of the nematic biofilm (Fig. 2a, Supplementary Fig. 4). To explore the physical mechanism underlying localization, we quantified the buckling condition. The localized buckling occurs just after reaching the critical chain length. As the chain continues to grow, extended arms follow the same localized buckling scenario (Fig. 2d, e).

The elasticity theory explaining the localization of the buckling is surprisingly complex. Both linear and nonlinear elastic properties of soft materials can contribute to the localization and formation of the post-buckling shape^29–34^. However, the theoretical framework for growth-induced localized buckling is not well studied. We have noticed that railroad thermal buckling^35^ and growing plant roots^36^ show a similar spatial localization profile. Therefore, we used the notation and the framework developed for these systems and followed the slender-body approximation to simulate the dynamics of a growing bacterial chain. Low-ambient temperature eliminates the twisting process; thus, we exclude the twisting dynamics, and we modeled the bacterial chain as an incompressible flexible elastic rod. Since the system is at low Reynolds number regime, we neglected the inertial forces and used the equations for mechanical equilibrium (see Supplementary Note 1 for the detailed derivations). First, we compared the buckling profiles of the growing rod under viscous drag force and uniform friction. We observed that the viscous drag force alone generates a global buckling profile. Similar global buckling shapes were also observed in growing liquid crystals^37^. However, uniform friction force provides a linear compressive tension profile which can localize the buckling. Further, we implemented a more realistic bacterial adhesive force model, recently developed by Duvernoy et al.^38^ to test the localization. In this model, interactions are based on local adhesion between bacteria and agar surface. Adhesive bonds are formed at a fixed rate and break when they experience the force larger than a threshold force. We also found that this model provides more localized tension profiles (Supplementary Fig. 5). Similarly, the tension locally exceeds the critical buckling limits, when the total length reaches the threshold. Our numerical simulations show that both uniform friction and adhesion models can create localized buckling. This buckling condition sets a length scale for a bacterial chain. The detailed comparison of the tension profiles is given in the Supplementary information.

### Finite-element simulation of growing biofilms

To further quantify the buckling conditions, we performed finite-element method (FEM) simulations based on recently developed algorithms used to study growing elastic structures^39–41^. In these simulations, we used uniform friction between a growing rod and a surface. Using realistic bacterial parameters, we found that an increase in friction force reduces the critical length (Fig. 2b, c). To experimentally verify this effect, we tuned the agarose concentration which serves as a flat surface and imaged the periodically buckled regions of a single-bacterial chain. Our experiments revealed that the critical length changes with the agarose concentration (Fig. 2d,e). Typically, the bacterial chain becomes unstable above 138 ± 26 μm. Interestingly, high-agarose concentration reduces the friction force and increases the critical length. The exact mechanism behind the friction^42^ is not clear to us; we speculate that the soft agar surface allows more local deformations and changes the local binding dynamics, which may increase the friction force. We also observed that the growth rate of the biofilm does not significantly change the critical length. This result suggests that the dynamics is overdamped by the friction (Supplementary Fig. 6).

Further, we tested the local buckling in a liquid environment, which eliminates the friction force. In its wild-type form, *B. subtilis* suppresses biofilm formation in liquid (LB) broth culture. Detailed molecular genetics studies have identified that protein *sinI* initiates and maintains the chaining state through double-negative feedback^1,3,4^ (Fig. 2f). To trigger biofilm formation in liquid, protein *sinI* should be overexpressed. To overcome this limitation, we used a genetically modified strain to drive *sinI* using IPTG (isopropyl-β-d-1-thiogalactopyranoside)-based induction (the background strains TMN1152 were received from R. Losick Lab). When we grew the biofilm in liquid culture with IPTG, local buckling disappeared, but the supercoiling process dominated the formation of the biofilm (Fig. 2g, Supplementary Movies 6 and 7). We further clarified the structure of supercoiled bundles using fluorescence time-lapse microscopy and SEM imaging (Supplementary Fig. 7). Experimentally, we observed variations in the shapes of early buckled bacterial chains (Supplementary Fig. 4). This variation is most likely originating from the surface heterogeneity. However, a localized buckling feature is conserved in all bacterial chains. Altogether, we showed that friction force between the agar surface and the bacterial chain controls the mechanical instability. Localization of stress further generates spatially localized buckling and defines the critical length scale for a biofilm.

### Edge instabilities

**Fig. 3:**
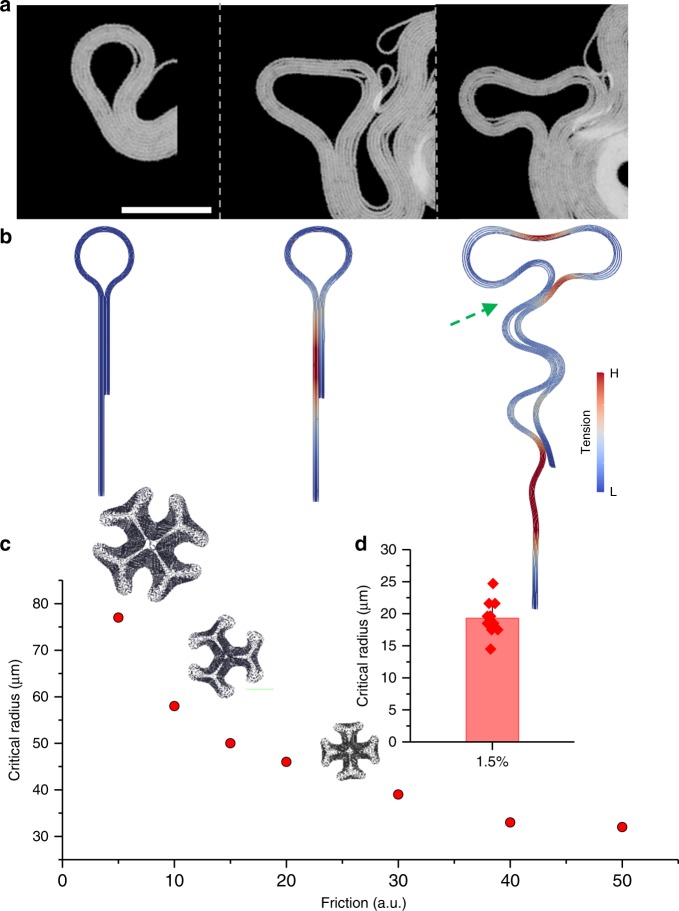
Edge instabilities of a growing biofilm. **a** Snapshots of growing multilayered circles. The radius reaches the critical radius and splits into two. Scale bar, 30 μm. **b** Snapshots from the simulation of a droplet-like structure with a long tail. Color code shows the stress level. While the droplet splits into two circles, the long tail locally buckles, since its length is above the critical buckling length. The green arrow shows the secondary buckling region which asymmetrically folds the circular structure onto the colony edge. **c** Multilayered growing circles are simulated under various friction forces. The plot of critical radius against friction, simulation results. Units are arbitrary. All snapshots were taken as the secondary edge instability occurred. **d** Experimental data of the critical radius. The colonies were grown on 1.5% agarose concentration. Error bar is defined as s.d.

As a second step, we focused on the edge instabilities of a growing biofilm. The growing multilayered circles are the most distinct geometric features observed around the leading edge (Fig. 3a). At first glance, the dynamics of these structures resemble the instabilities of smectic-A liquid crystal filaments^37,43^. These circles are connected to the film with a tail, and they grow like droplets. As they grow outward, instability occurs, and the droplets split into two. As gleaned from previous studies, we tested whether these structures have a characteristic critical radius which triggers new mechanical instabilities. We performed FEM simulations to observe the splitting dynamics. First, we simulated the growing multilayered circular structure with a tail (Fig. 3b, Supplementary Movie 8). We found that these structures indeed have a critical radius. Above this radius, circular structures are unstable and split into smaller but stably growing ones. As we observed in previous experiments, the straight tail also locally buckles in the direction perpendicular to the growing axis after reaching the critical length. Our simulation verified that edge instability occurs when the circumference of the circular structure exceeds the linear critical length $2{\mathrm{\pi }}R_c = \ell _c$ (Fig. 3c, d). We confirmed this relationship experimentally. Similarly, both a critical radius and a critical length are controlled by friction.

We also observed that, just after the splitting, an additional buckling might occur around the junction point (connection between the droplet and the tail) where the chain has the highest curvature (green arrow in Fig. 3b). This buckling further makes the divided droplet fall back on the biofilm edge asymmetrically (Supplementary Fig. 8). We then extended our simulations to observe large-scale biofilm growth starting from many concentric circular rods or multilayered structures (Supplementary Movies 9 and 10). In our simulations, initial filament configurations can also affect the final colony shape. A U-shaped multilayered structure gives a more realistic colony morphology (Supplementary Figs. 9 and 10). As expected, friction also shapes the overall biofilm morphology by defining the maximum radius of the curvature around the leading edges (Fig. 3d). Altogether, our results show that a growing biofilm has a critical radius which controls the dynamics of edge instabilities. It should be noted that the instabilities of a biofilm structure are mainly defined by the competition between elastic deformation of a growing rod and the rod-surface interactions. Friction or adhesion forces are responsible for the rod-surface interactions and control the instabilities. In addition to the friction force, the elastic modulus of the filament can directly change the buckling conditions (Supplementary Fig. 11).

### Dynamics of topological defects

**Fig. 4:**
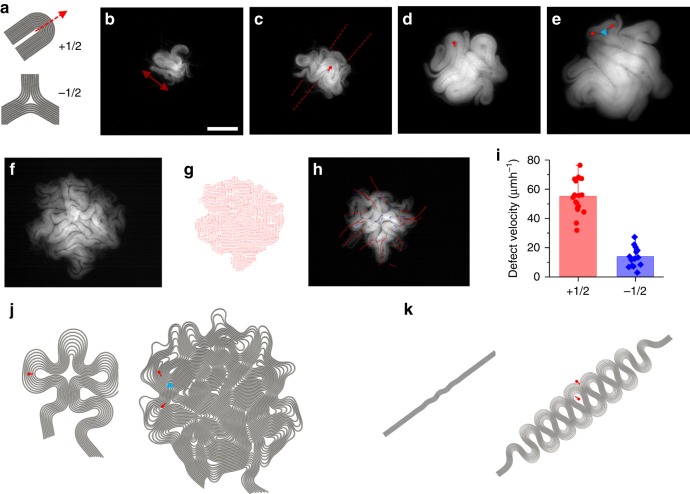
Dynamics of topological defects. **a** Comet-like motile (+1/2) and trefoil-like (−1/2) topological defects. **b**–**e** Snapshots of a growing biofilm. **b** The biofilm elongates and reaches the critical buckling length. Scale bar 100 μm. **c** As a result, the biofilm shows a structural folding and creates (+1/2) topological defects. **d**, **e** In total, (+1/2) topological defects at the edge move outward, split into two (+1/2) defects, and create a (−1/2) defect at the junction. **f** Snapshot of a growing biofilm. **g** The director field of the biofilm (**f**). **h** Trajectories of both (+1/2) and (−1/2) defects. Red lines indicate the trajectories of (+1/2) defects which are motile, whereas blue lines represent the trajectories of (−1/2) defects, which are stationary. **i** Experimental data of velocity distribution of (+1/2) and (−1/2) defects. Error bars are defined as s.d. **j**, **k** Simulation results of growing multilayered structures. **j** In total, the (+1/2) defect splits and forms paired (+1/2 red dots) and (−1/2 blue dot) defects. **k** Large colony folding forms only (+1/2) defects (red dots)

One of the characteristics of active nematics is the formation of topological defects^16–18,27,28,44^. To better understand the active matter nature of a biofilm, we further focused on how mechanical instabilities generate topological defects. In microtubule-based active matter systems, topological defects (+1/2 or −1/2) (Fig. 4a) are spontaneously created and annihilated. The exact mechanism behind the creation of these defects remains unknown^45^. Recent studies elegantly demonstrate that the confinement controls defect formation^45–47^. In contrast, our biofilm system does not require any physical confinement which enables the edge to move freely and allows observation of the evolution of the defect formation. By analyzing the development progress, we found two main defect-forming mechanisms: the large-scale biofilm folding and edge instabilities.

Using time-lapse microscopy, we recorded the first defect generation process. As it grows, the entire biofilm reaches the critical length scale and triggers a large structural folding (Fig. 4b, c). This folding forms +1/2 defects. Figure 4 shows the temporal evolution of this defect formation. As observed, the biofilm sequentially folds in orthogonal directions and generates several motile +1/2 defects (Supplementary Fig. 12). Clearly, in this stage, the total number of defects increases. As the size of a biofilm increases, the critical length drops below the biofilm size, and this type of folding appears locally (Supplementary Movies 2 and 3). In bulk active matter systems, defects are generated in pairs. However, active matter systems with boundaries can generate the +1/2 defect, penetrating into the system from the edge. Figure 4j, k shows the simulation results representing this unconventional defect-forming mechanism.

We also found that the edge instabilities can be considered as a second type of defect-forming mechanism, which is often observed in bulk active matter systems. Growing droplet geometries around the edge simply behave like +1/2 topological defects (Fig. 4d). These structures are very motile, because the unbalanced force generated by the growing side chains can push the defect forward. In contrast to the structural folding described above, this growing droplet splits into two and generates paired +1/2 defects, and it leaves one −1/2 defect around the junction point (Fig. 4e, j). This mechanism keeps the total topological charge constant. At later stages, foldings and splittings become strongly coupled. Thus, the dynamics of defect formation become very chaotic.

Our simulations verified the dynamics of defect formation around the edge. We observed that above the critical radius, both outward- and inward-propagating bucklings appear. Accumulated localized stress gradually slows down the speed of the central region and then starts migrating inward. These inward-propagating chains might collide with the junction point and then become a stationary −1/2 defect by leaving two motile paired +1/2 defects (Supplementary Movies 9 and 10).

Notably, some of the propagating defects leave king bands as a clear trace (Fig. 4f). These bands originate from the differences in the radius of multilayered structures which eventually define the total growth rate of the circumference.

To further examine defect formations experimentally, we measured the speed distributions of defects within a biofilm (Fig. 4i). We found that +1/2 defects are more motile around the edge and −1/2 defects are stationary. We also noticed that as the biofilm develops, the central region becomes stationary. We measured the GFP signal around the center and noticed that the growth of the biofilm does not slow down (Supplementary Fig. 13). The most parsimonious hypothesis based on these observations is that stress accumulates around the center and slows down the dynamics.

### Defect-mediated stress and formation of sporulation sites

**Fig. 5:**
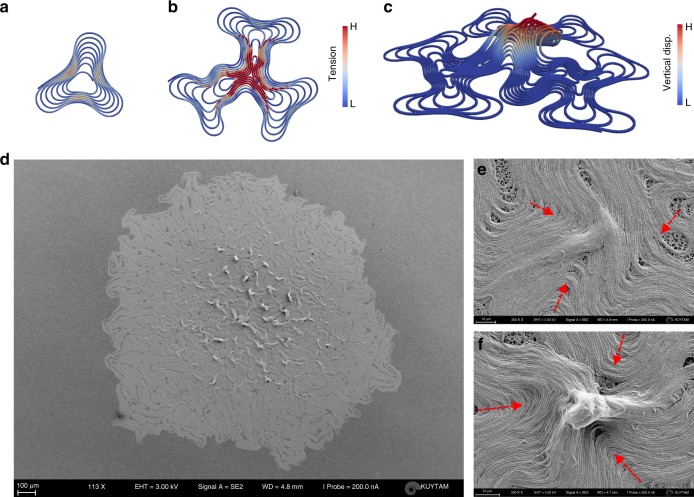
Accumulation of stress and vertical liftoff. **a**–**c** Simulation results of a growing multilayered circular colony. **a** After reaching the critical radius, the colony folds and creates topological defects. **b** Inward-propagating defects collide and create a (−1/2) defect. The stress is accumulated at the collision point, which is a (−1/2) topological defect. **c** The accumulated stress results in vertical lifting around the (−1/2) topological defect. **d**–**f** SEM images of a biofilm in the late stage. Red arrows indicate the propagation directions of the defects. **d** Vertically lifted domains are in the vicinity of the center where the stress accumulates. **e** Motile (+1/2) defects collide and form a stationary (−1/2) defect. **f** Accumulation of stress causes vertical liftoff

Topological defects could also change the physiology of the biological tissues^18,48–50^. It was shown that defect-induced stress triggers mechanotransduction and alters the gene expressions^16^. Stress also modulates the cellular density of cultured cells. Topological defects and active stress could also play a biophysical role during the development of a biofilm. With this motivation, we further performed stress analysis of topological defects. Our simulations revealed that the stress accumulates as a stationary −1/2 defect occurs (Fig. 5a, b). Around these defects, the unbalanced force generated by the growing chain can be balanced by counterpropagating ones. Further, colliding bent chains significantly increase total stress. When we performed our simulation in the three-dimensional domain (so far we performed all simulations in a 2D confined environment), we observed vertical lifting of the elastic rods in the vicinity of −1/2 defect (Fig. 5c, Supplementary Movie 11).

We asked whether we could experimentally observe the effects of stress accumulation in a biofilm. To do so, we analyzed the 3D surface topology after getting a stable biofilm (35 h after seeding). SEM images revealed vertical structures around the −1/2 defects sites (Fig. 5d–f). A close investigation clarified that these structures are actually the vertically lifted parts of the defects. These structures are more visible around the center of the biofilm. We grew the non-chaining bacteria in the same conditions and we did not observe any of these vertical structures (Supplementary Fig. 14). Isolated bacterial colonies can form multilayered structures but do not form a vertical structure (Supplementary Fig. 15). The results are remarkably consistent with our FEM simulations. These structures have already been identified as aerosol or fruiting bodies of the biofilm, which eventually produce bacterial spores^2^. We do not exclude other structural instabilities or the contribution of the twisting process. Our results support the idea that topological defects are one of the possible origins of these vertical-ordered geometries. Finally, we tested the instabilities of growing *E. coli* biofilm structure^51^. A colony of isolated *E. coli* provides a smooth leading edge. However, biofilm-forming *E. coli* also shares similar buckling and edge instabilities (Supplementary Fig. 16).

## Discussion

The theory of active nematics provides a robust framework to explain the coordinated motion of cells and the emergence of large-scale order in biological systems. Our study throws light upon the mechanical aspects of biological active matter systems: first, mechanical instabilities can be simply characterized by a critical length scale. Different types of length scales have previously been defined for various AN systems^24,26^. These quantities mainly describe the activity of the system. In contrast, our length scale reflects the elastic properties of the biofilm, which is mainly defined by the competition between elastic deformation of chaining bacteria and the bacteria–agar interactions. Second, the leading edge of the biofilm is not stable. Edge instabilities^15,44,52–54^ have received significant attention due to fingering formation during wound healing^15^. Our results revealed the importance of cellular alignment and localized stress-driving instabilities. Finally, we have shown the formation of the defects and their effect on the stress distribution across the biofilm. In growing tissue or bacterial colonies, mechanical stress can easily result in physiological stress. Recent studies have linked the physical forces with the cellular response^16^, and our system could further reveal the importance of stress management across growing tissue^55^. Another benefit of using *B. subtilis* is the availability of a sophisticated genetic toolbox^56^. Genetic manipulations and control could reveal unexplored dynamics of active matter systems. We believe that our biofilm platform offers new opportunities to study active nematics in living systems.

## Methods

### Sample preparation and growth conditions

Bacterial cultures (BAK47 and BAK51) were grown in LB broth at 37 °C on a shaker. An overnight culture was diluted 100× and grown for 8 h. The culture was diluted 10,000×, and 50 µl of culture was seeded on an LB agarose plate. These isolated bacteria on plates were grown at 21 °C for 12 h and then imaged. The cover glass was not used during imaging. IPTG (isopropyl-β-D-1-thiogalactopyranoside)-inducible bacteria (BAK50) were grown in LB broth at 37 °C without IPTG induction to ensure that chaining does not occur. An overnight culture was diluted 10,000× and supplemented with 100 μM IPTG. A droplet of IPTG-inducible bacterial suspension was placed between two glass slides with 100-µm separation, and then imaged at 21 °C. Strains used in experiments are described in Table 1.

**Table 1:** Strain list used in this study

Strain	Parent	Operation	Genotype
BAK47	168	Transformed with plasmid ECE321 from Bacillus Genetic Stock Center	*amyE::*Pveg-sfGFP (Spc)
BAK50	TMN1152	Transformed with plasmid ECE321 from Bacillus Genetic Stock Center	*amyE::*Pveg-sfGFP (Spc) *ywrK*::P_spank_-*sinI* (Spc)
BAK51	TMN1138	Transformed with plasmid ECE321 from Bacillus Genetic Stock Center	*amyE::*Pveg-sfGFP (Spc) *sacA*::P_hag_-mKate2L (Kan) *hagA233V* (Phleo)
BAK55	DH5alpha	Transformed with plasmid 107743 from Addgene	*pBad-ag43, pDawn-sfGFP*

### *E. coli* biofilm formation

*E. coli* bacterial cultures (BAK55) were grown in LB broth at 37 °C on a shaker. An overnight culture was diluted 100× and grown for 8 h. The culture was diluted 10,000×, and 50 µl of culture was seeded on YESCA plates and were grown at 37 °C. On regular LB plates and at room temperature, *E. coli* does not form a biofilm.

### Microscopy imaging

Fluorescence time-lapse imaging was performed using a Nikon SMZ18 stereomicroscope, and images were obtained using a Thor Labs DCC1545M CMOS camera. Time intervals between successive images are 15 min. To obtain the best image quality, the light exposure was controlled adaptively for different colony sizes by a custom-written program in LabVIEW. A typical colony growth experiment was run for 1 day.

### SEM imaging

Before seeding the bacteria on LB agar surface, a sterile filter paper with a pore size of 0.2 µm was placed on the surface. After seeding the bacteria on the filter paper, bacteria were grown on the paper for 24 h at 21 °C. Then the filter paper was peeled off from the surface, and the colonies were fixed using paraformaldehyde and left to drying. Fixed colonies were imaged using a Zeiss Ultra Plus Field Emission Electron Microscope.

### Image analysis and detection of topological defects

Images were smoothed using Bandpass filter in ImageJ, and Coherence-Enhanced Diffusion Filter was applied to images in MATLAB. The ImageJ plugin OrientationJ was used to find the nematic director field of the colony. We followed the defect detection algorithm^27^ using a custom-written MATLAB code.

### Computer simulations

For the computer simulations involving large growth, we employed a dynamic finite-element program^39–41^. The filament was modeled as an isotropic, linearly elastic continuum with three-dimensional beam theory, using cubic Hermite-shape functions and a corotational quaternion formulation for geometric nonlinearity. The filament was assumed to have a circular cross section with constant uniform radius of *r* = 0.7 μm, a Young’s modulus of *E* = 5300 Pa (corresponding to a bending stiffness of *E*(π/4)*r*^4^ = 10^−21^ *Nm*^2^), a Poisson ratio of 1:3, and a mass density of 1 g cm^−3^. The elastic energy of the filament consisted of the sum of bending, torsion, and axial compression energies. Hertzian repelling forces were exchanged between contacting filament elements in normal direction, whereas the tangential contact forces were realized with a Coulomb slip-stick friction model. Newton’s equations of motion were integrated in time with a second-order predictor–corrector scheme. For numerical robustness and to keep the filament near static equilibrium during growth, subcritical damping forces were added. The initial configuration was assumed to be stress-free, and the filament was grown exponentially in length over time, according to $\ell (t) = \ell _0e^{\sigma t}$ where *σ* is the exponential growth rate. This was realized by continuously increasing the equilibrium length of each rod element. The substrate was modeled as an ideal elastic horizontal plane onto which the filament was placed, and a gravitational force was applied perpendicular to the plane.

## Supplementary information

Supplementary Information

Description of Additional Supplementary Files

Supplementary Movie 1

Supplementary Movie 2

Supplementary Movie 3

Supplementary Movie 4

Supplementary Movie 5

Supplementary Movie 6

Supplementary Movie 7

Supplementary Movie 8

Supplementary Movie 9

Supplementary Movie 10

Supplementary Movie 11

## Data Availability

All data supporting the findings of this study are available from the corresponding authors on request.

## Code availability

All custom codes used in this study are available from the corresponding authors on request.
