# Supplementary material for: Emergence of active nematics in chaining bacterial biofilms
Source: Nat Commun. 2019 May 23;10:2285. doi: 10.1038/s41467-019-10311-z (PMC6533293; doi:10.1038/s41467-019-10311-z)
Supplement: Supplementary file 1 — Supplementary Information [file 41467_2019_10311_MOESM1_ESM.pdf]

# Emergence of active nematics in chaining bacterial biofilms
# Supplementary Information

Yaman et al.

## Supplementary Figures

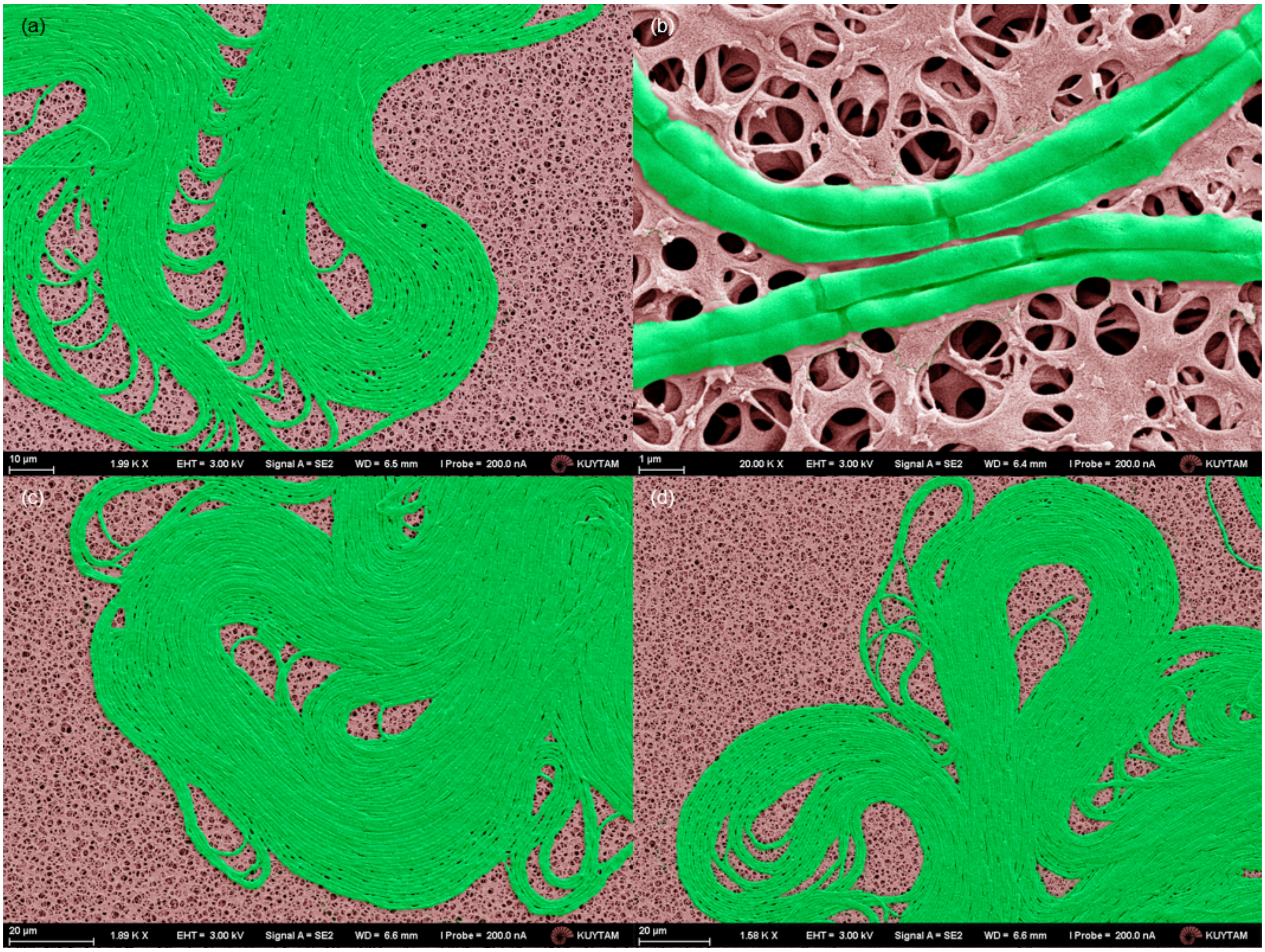


Supplementary Figure 1: **SEM images of bacterial biofilm (*BAK47*).** (a), (b), (c), (d) SEM images of chaining bacteria on a filter paper. Circular multilayered structures, aligned chains, and kink bands appear during biofilm growth.

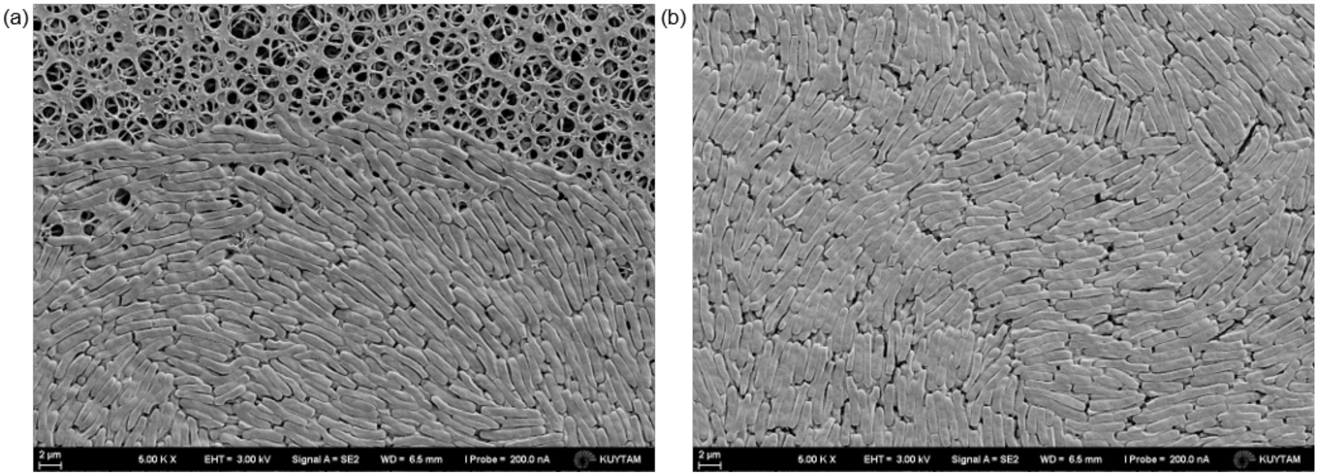


Supplementary Figure 2: **SEM images of the non-chaining bacterial strain (*BAK51*).** (a) Tangentially oriented cells form a smooth boundary layer. (b) Cells are nematically oriented and tightly packed.

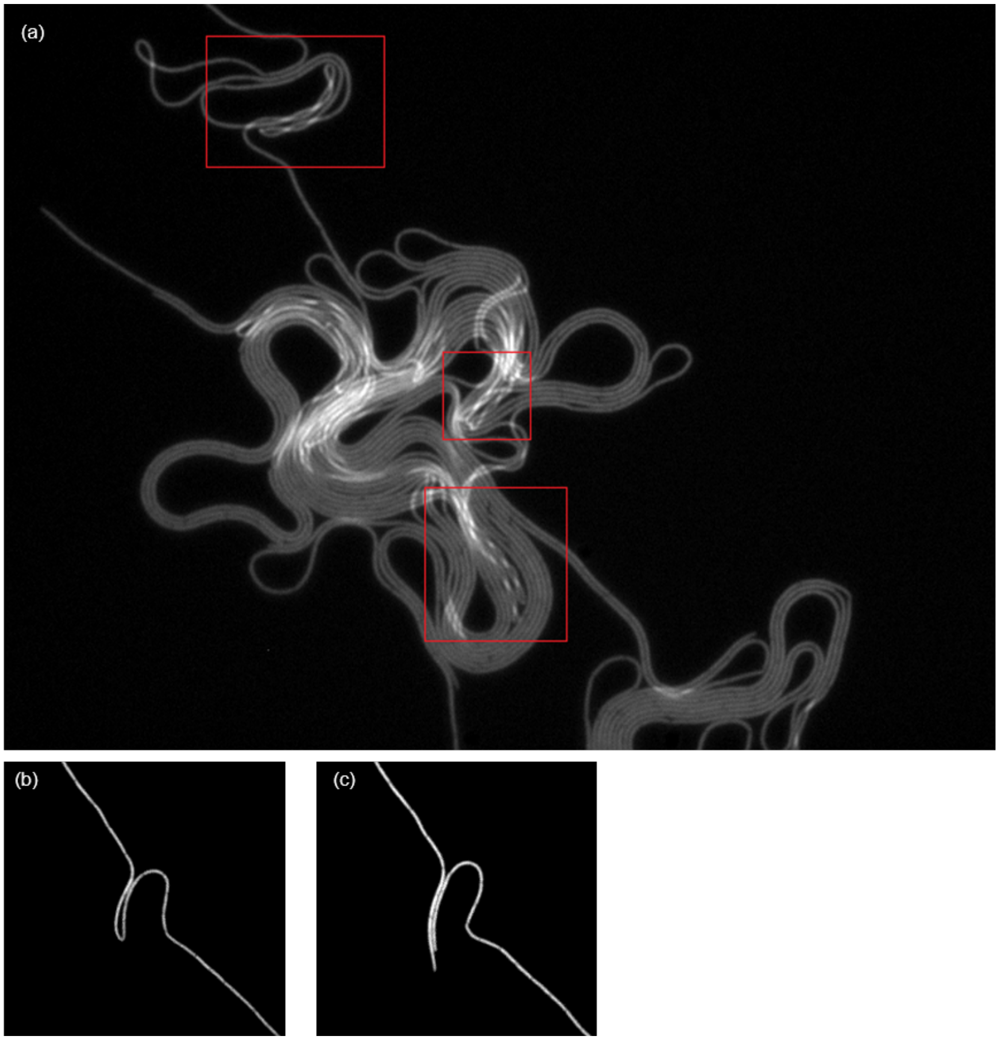


Supplementary Figure 3: **Twisting and breakage of a bacterial chain.** Images of a growing biofilm at $37^oC$. (a) Supercoiled structures are indicated with red boxes. (b), (c) Snapshots are showing the breakage of a buckled chain during growth.

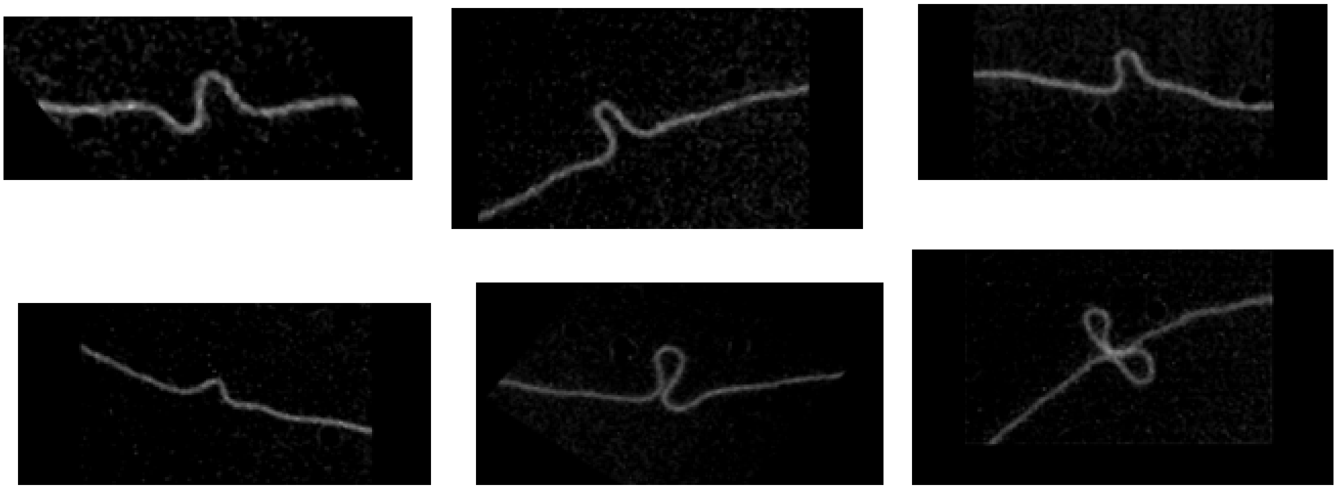

Supplementary Figure 4 **Early buckling shapes of the growing bacterial chains.** Surface heterogeneity creates different buckling shapes. However, all of them share the same localized profile.

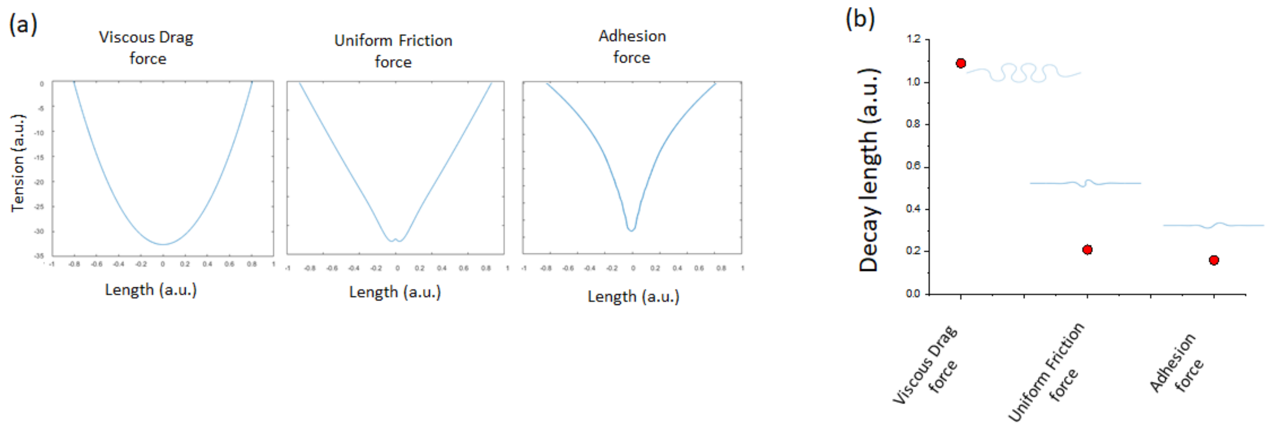


Supplementary Figure 5: **Global versus local buckling.** Viscous drag force generates global buckling. (a) Simulation of tension and buckling profiles of growing rods under different forces; viscous drag, uniform friction and adhesion-based model. (b) Decay lengths of the buckling profiles under different friction force models. Both, uniform friction and adhesion model can localize the buckling around the center.

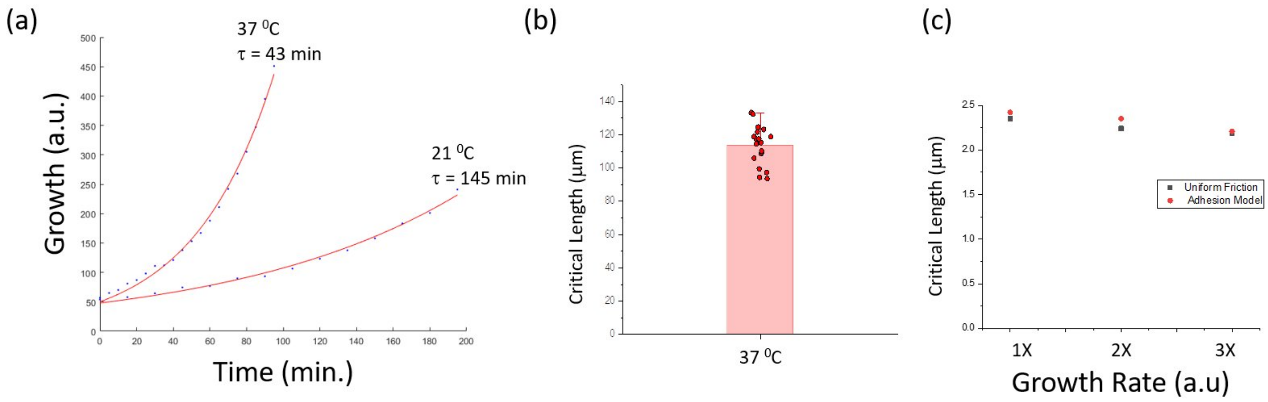


Supplementary Figure 6: **Temperature dependence of biofilm formation.** (a) Growth curves of the biofilms at different ambient temperatures. (b) Distribution of the critical length measured at $37^oC$. We observed a small decrease in the critical length. Error bar is defined as s.d. (c) Numerical simulation of critical length as a function of growth rate for both uniform friction and adhesion models.

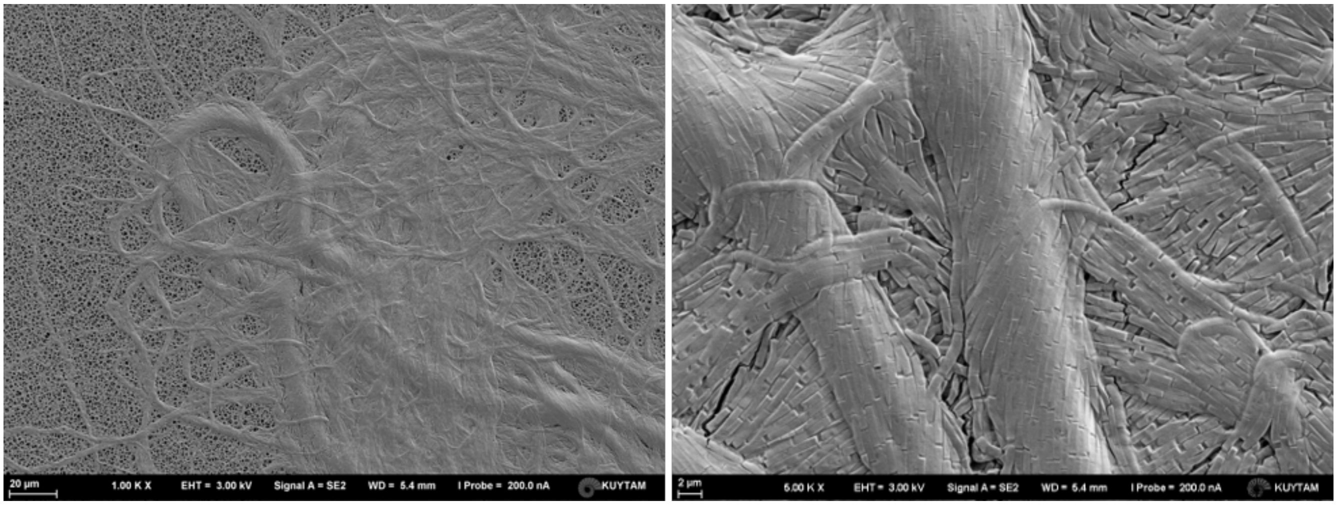


Supplementary Figure 7: **SEM images of supercoiled bundles.** Strain *BAK50* was grown in liquid LB culture.

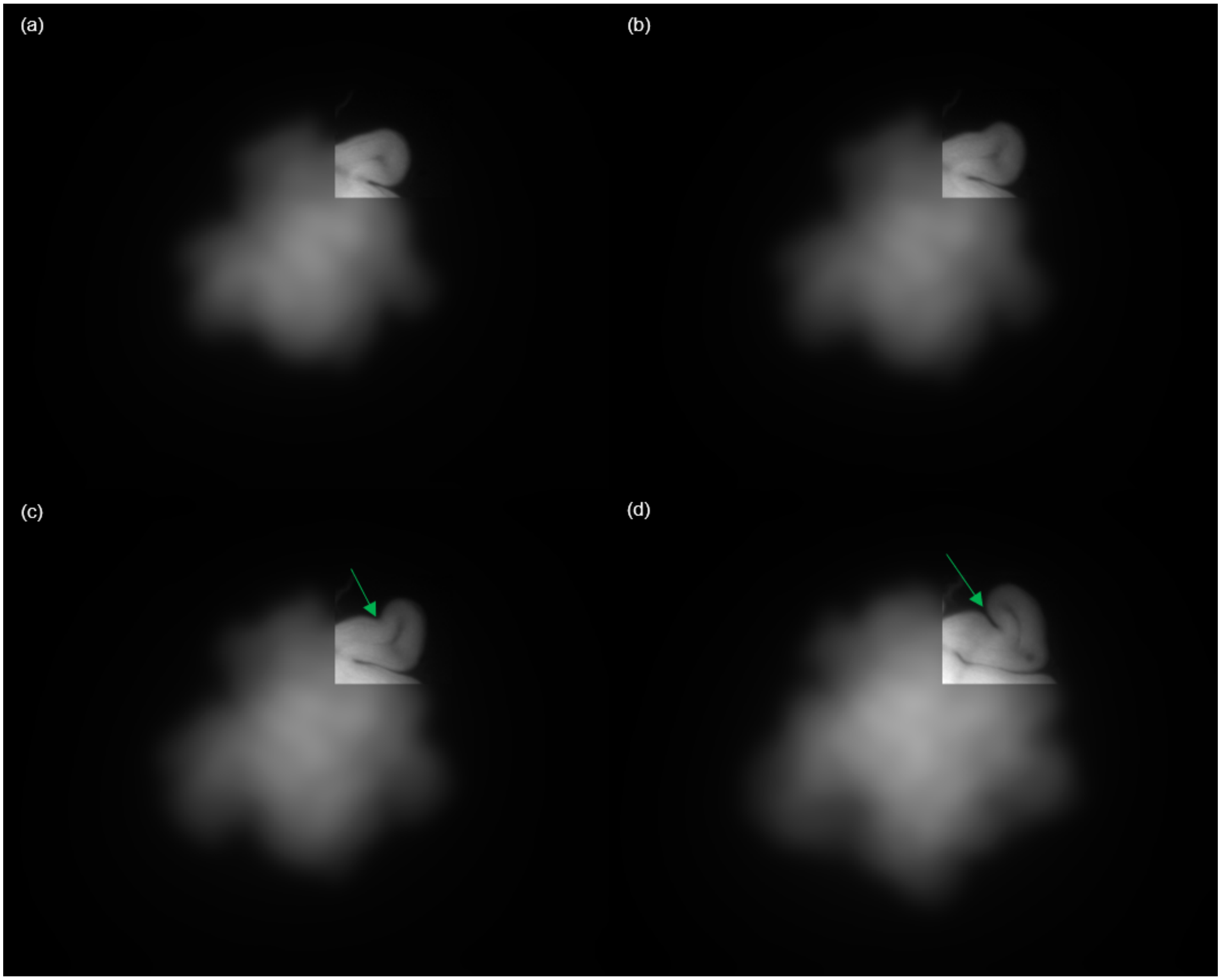


Supplementary Figure 8: **Asymmetric splitting of a droplet-shaped structure.** (a), (b), (c), (d) Snapshots from the biofilm on 1.5% agar surface. Green arrows show the additional buckling creating asymmetric splitting of the defect.

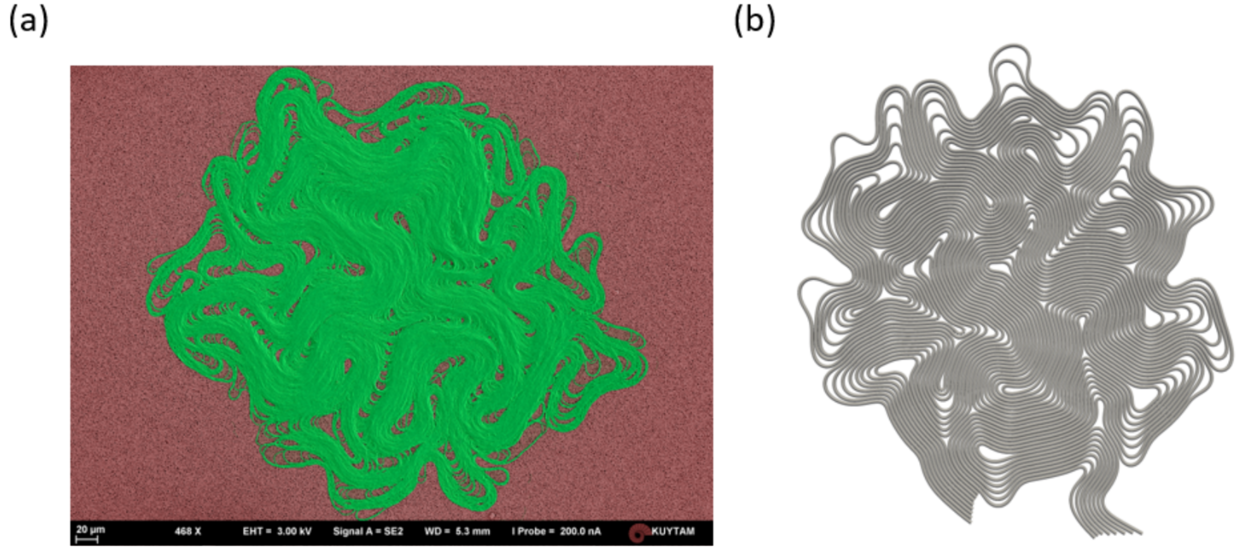


Supplementary Figure 9: **Comparison of a real and simulated chaining bacterial biofilms.** (a) SEM image of a biofilm. (b) Snapshot from the simulation.

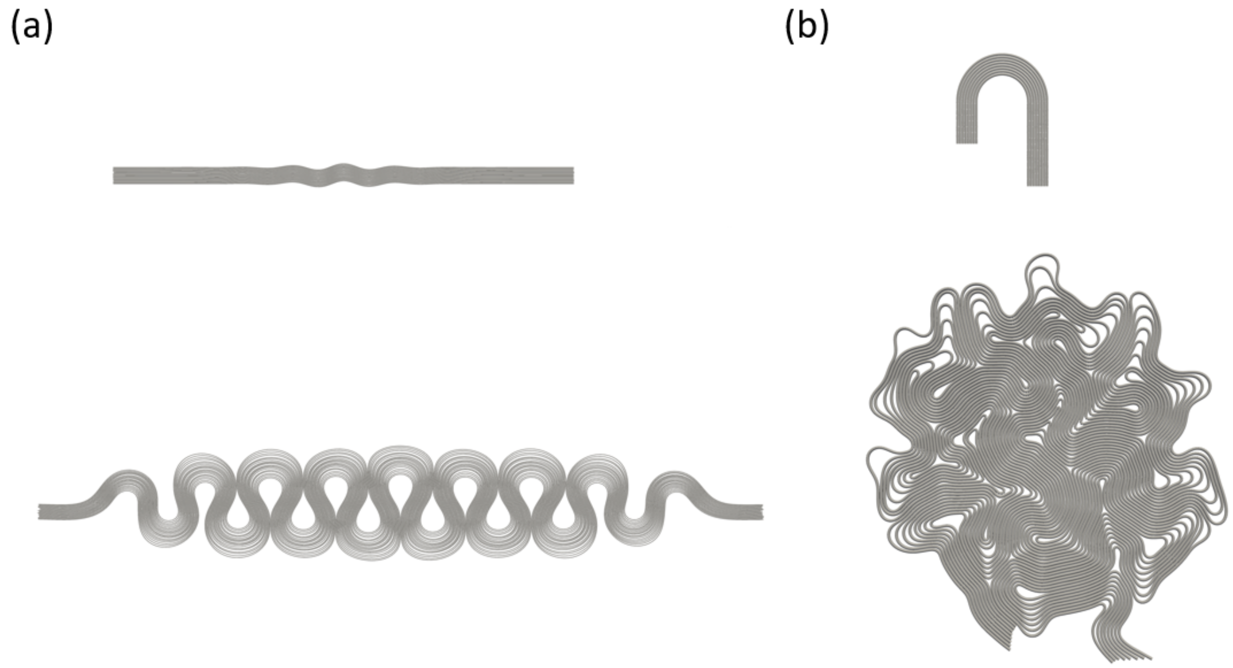


Supplementary Figure 10: **Simulation of growing colonies starting from different initial shapes.** (a) Linear and (b) U-shaped multilayered structures used as initial shapes. U-shaped initial configurations give rise to circular colony observed in the experiments.

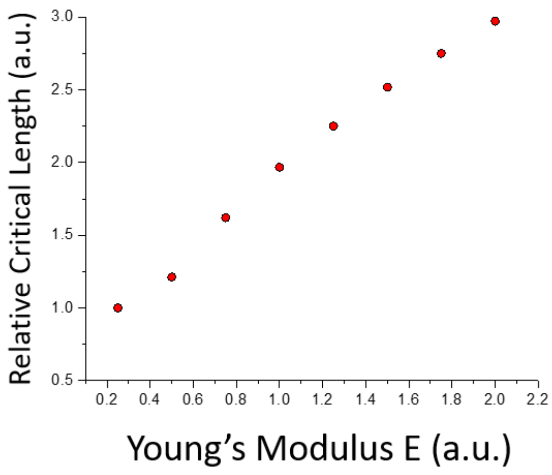


Supplementary Figure 11: **Simulation of relative critical length as a function of Young’s modulus of a growing rod.** The critical length increases with the higher Young’s Modulus values.

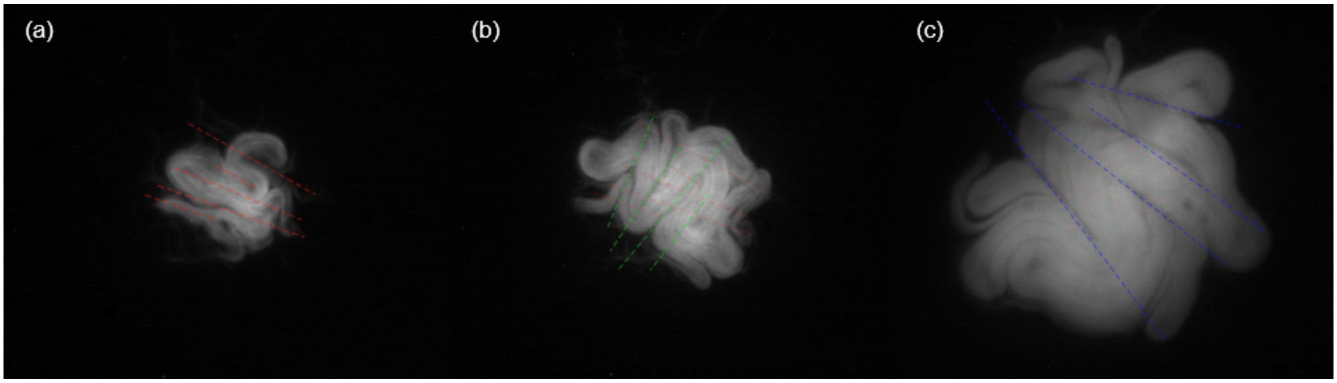


Supplementary Figure 12: **Large-scale structural folding of a biofilm.** Defect-creating large-scale structural buckling mechanism is observed for the biofilms at the size above the critical length. (a), (b), (c) show successive folds in orthogonal directions. Colored lines show the propagation directions of the topological defects.

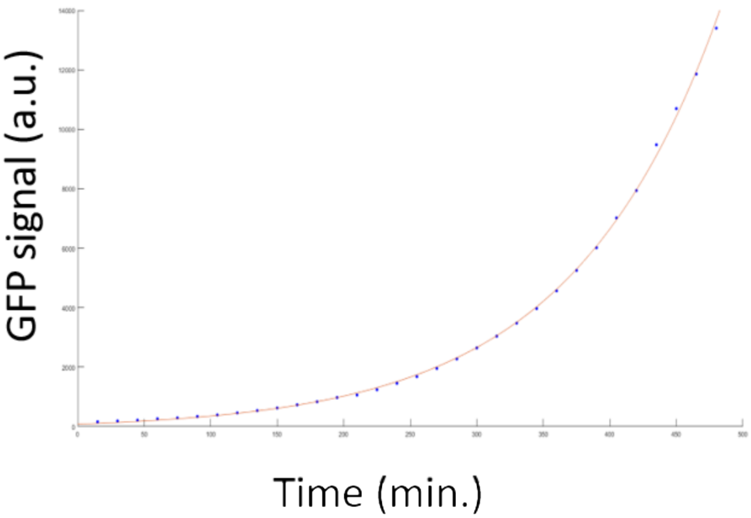


Supplementary Figure 13: **Growth curve of the biofilm.** GFP signal as a function of time shows normal growth profile. Time constant was measured to be $t = 150$ min at room temperature.

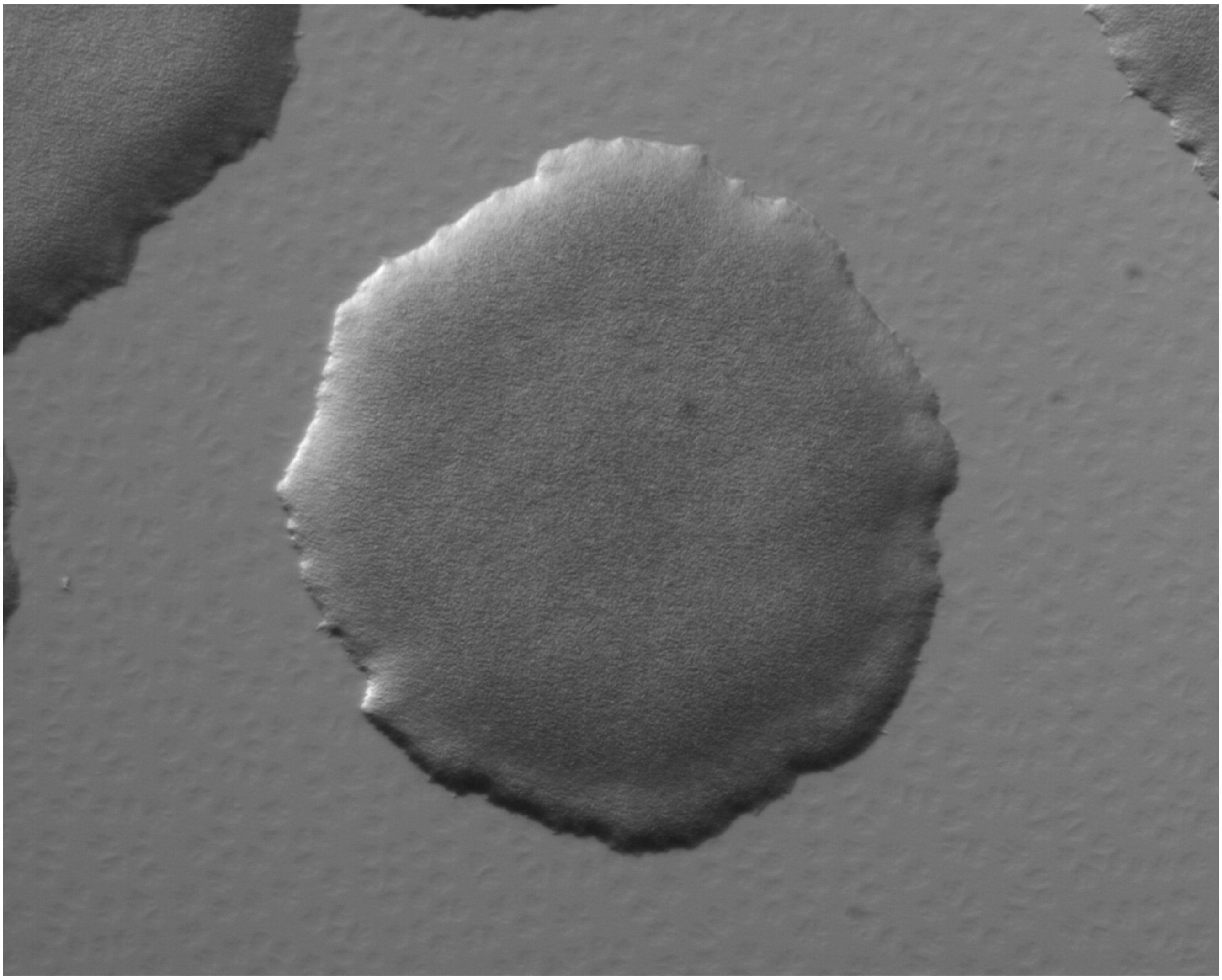

Supplementary Figure 14: **The isolated bacterial colony of non-chaining *B. subtilis* strain (*BAK51*).** Bright-field image of the colony grown on 1.5% agar surface. Vertical fruiting bodies not observed.

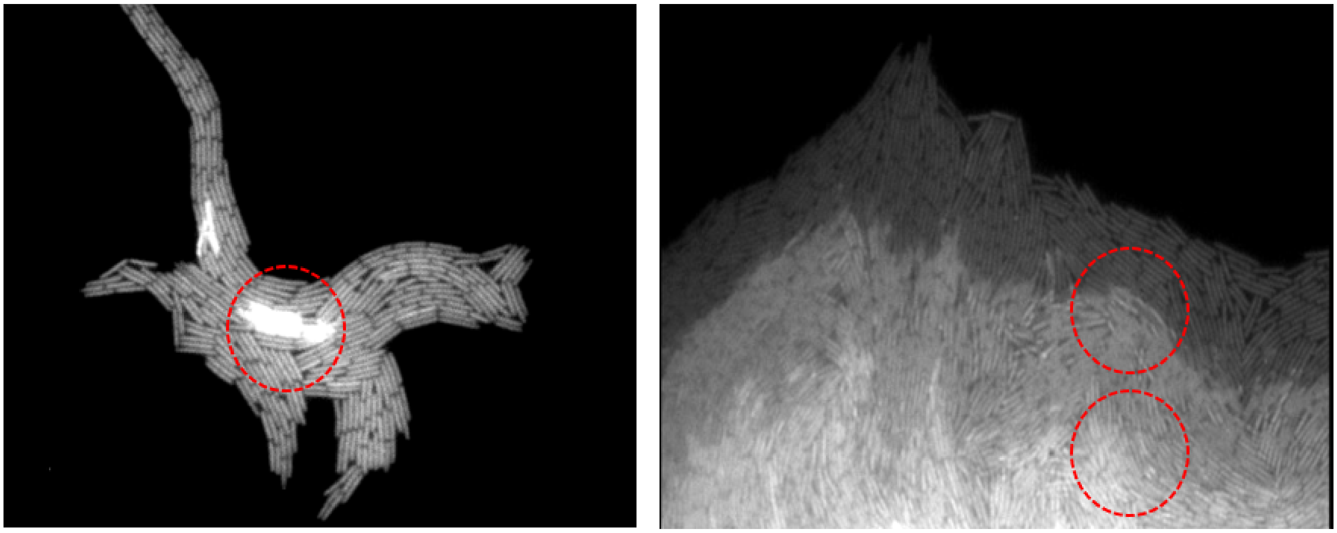

Supplementary Figure 15 **Double and multilayer formation in isolated bacterial colonies.** Red circles indicate the regions. Instead of forming vertical structures, these layered regions gradually cover the colony. The transition can be observed around the edge.

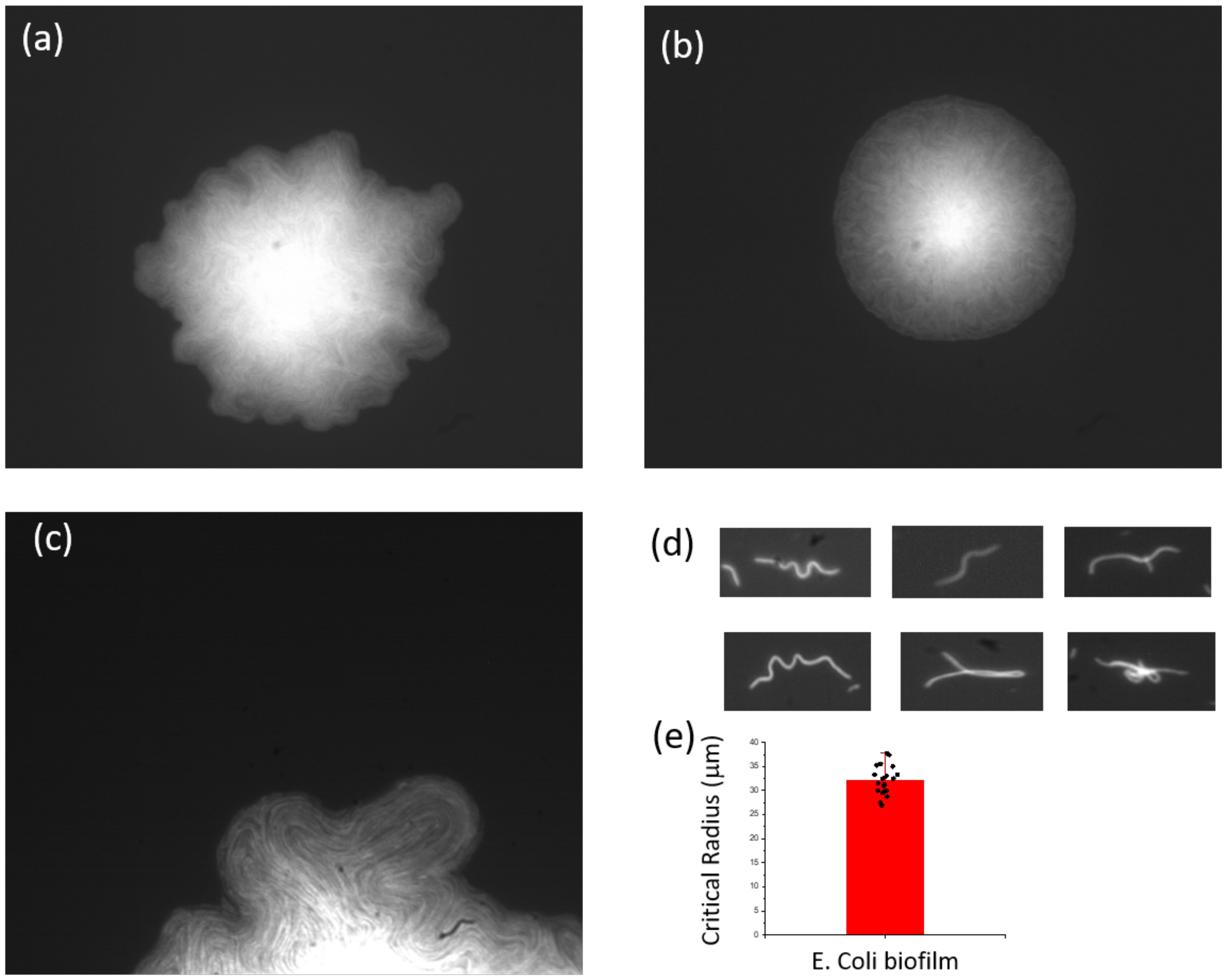


Supplementary Figure 16 **Edge instabilities and local buckling of *E. coli* biofilm.** a) Biofilm forming *E. coli* on YESCA plates forms strong edge instabilities. b) *E. coli*, growing on regular agarose (LB) plate form smooth colony edge. c) Similarly, above the critical radius circular growing patterns split. d) Sample images of early buckling shapes of chaining *E. coli*. e) Distribution of critical radius of the *E. coli* biofilm. Error bar is defined as s.d.

**Supplementary Table 1: The parameters used in the simulations based on adhesion model**

| | |
|---|---|
| $\nu = 1$ | Drag coefficient |
| $EI = 1$ | Bending modulus |
| $\ell = 1$ | Initial length of the rod |
| $\sigma = 100$ | Exponential growth rate |
| $k_{on} = 5e4$ | Adhesin attachment rate |
| $k_{off} = 2$ | Adhesin detach rate |
| $L_0 = 0.05$ | See eqn. (13) |
| $A = 60$ | See eqn. (13) |
| $F_{link}$=60 | See eqn. (14) |
| $n = 50$ | Max. number of adhesins per node |

## Supplementary Note 1: Numerical simulations of a growing rod

The equation of motion describing the dynamics of an inextensible growing rod under external forces in a viscous environment is:

$$\nu \mathbf{x}_t = -EI\mathbf{x}_{ssss} + \mathbf{f_{ext}} + \partial_s(T\mathbf{x}_s) \tag{1}$$

Here, $\nu$ is the local drag coefficient of the fluid, $EI$ is the bending modulus of the rod and $T$ is the tension on the rod. Subscripts $s$ and $t$ denotes the differentiation with respect to arclength parameter and time respectively. The growth condition for the exponentially growing rod is:

$$\mathbf{x}_s\mathbf{x}_{st} = \sigma \tag{2}$$

Where $\sigma$ is the exponential growth rate of the rod. Differentiating eqn. (1) with respect to arclength gives:

$$\nu \mathbf{x}_{st} = -EI\mathbf{x}_{sssss} + \partial_s\mathbf{f_{ext}} + \partial_{ss}T\mathbf{x}_s + T\mathbf{x}_{sss} + 2\partial_s T\mathbf{x}_{ss} \tag{3}$$

By multiplying both sides with the unit tangential vector we get:

$$\nu \mathbf{x}_s\mathbf{x}_{st} = -EI\mathbf{x}_s\mathbf{x}_{sssss} + \mathbf{x}_s\partial_s\mathbf{f_{ext}} + \partial_{ss}T\mathbf{x}_s\mathbf{x}_s + T\mathbf{x}_s\mathbf{x}_{sss} + 2\partial_s T\mathbf{x}_s\mathbf{x}_{ss} \tag{4}$$

By using the following identities and eqn. (2), we have:

$$\begin{gathered}\mathbf{x}_s\mathbf{x}_s = 1;\ \mathbf{x}_s\mathbf{x}_{ss} = 0;\ -\mathbf{x}_s\mathbf{x}_{sssss} = 4\mathbf{x}_{ss}\mathbf{x}_{ssss} + 3\left|\mathbf{x}_{sss}\right|^2;\ \mathbf{x}_s\mathbf{x}_{sss} = -\left|\mathbf{x}_{ss}\right|^2 \\ \left(\partial_{ss} - \left|\mathbf{x}_{ss}\right|^2\right)T = 4EI\mathbf{x}_{ss}\mathbf{x}_{ssss} + 3EI\left|\mathbf{x}_{sss}\right|^2 - \mathbf{x}_s\partial_s\mathbf{f_{ext}} + \nu\sigma\end{gathered} \tag{5}$$

Numerical solution of eqn. (1) and eqn. (5) leads to the time evolution of a growing rod under external forces in a viscous fluid. In our case, we solved this equation for viscosity dominated, uniform friction dominated, and adhesion dominated system. For the viscous dominated system, the external force is zero. For the other two cases, we replaced $\mathbf{f_{ext}}$ with uniform friction force or adhesion.

### a. Viscous Drag Force Dominated System

First, we assume that viscous forces are dominating the filament dynamics and we analyzed the buckling shape of a growing rod. Therefore, we neglected all other external forces. Then the eqn. (5) becomes:

$$\left(\partial_{ss} - \left|\mathbf{x}_{ss}\right|^2\right)T = 4EI\mathbf{x}_{ss}\mathbf{x}_{ssss} + 3EI\left|\mathbf{x}_{sss}\right|^2 + \nu\sigma \tag{6}$$

For a straight rod, $\mathbf{x}_{ss} = \mathbf{x}_{sss} = \mathbf{x}_{ssss} = 0$ for any point. Therefore, equation (6) reduces to:

$$\partial_{ss}T = \nu\sigma \tag{7}$$

The solution of this equation with free end boundary conditions is a parabola:

$$T = -\nu\sigma\left(\frac{\ell - x}{2}\right)^2 \tag{8}$$

Here $x$ is the position of a point on the straight rod and $\ell$ is the total length of the rod. The simulation of this condition are given in Supplementary Figure 1.

### b. Uniform Friction Force Dominated System

To find the tension profile of this system, we neglected the viscous forces and assuming that the center of the rod is positioned at $x = 0$, we can write the external forces as:

$$\mathbf{f_{ext}} = -f sgn\left(x\right)\mathbf{x}_s \tag{9}$$

Where $f$ is the friction per unit length. By substituting $\mathbf{f_{ext}}$ into eqn. (5), we get:

$$\left(\partial_{ss} - \left|\mathbf{x}_{ss}\right|^2\right) T = 4EI\mathbf{x}_{ss}\mathbf{x}_{ssss} + 3EI\left|\mathbf{x}_{sss}\right|^2 - \mathbf{x}_s\partial_s\mathbf{f_{ext}} + \nu\sigma \tag{10}$$

We neglect the viscous term and by assuming the rod is straight and we get:

$$\partial_{ss}T = \partial_s f sgn\left(x\right) \tag{11}$$

The solution of this equation with free end boundary conditions provides a linear tension profile:

$$T = f\left(|x| - \ell/2\right) \tag{12}$$

### c. Adhesion model

To implement the adhesion force, we used a recently developed model in which adhesive proteins dynamically attach and detach to the agar surface and the force is obtained by using the worm-like-chain model[1]. In our model, adhesins can be uniformly distributed along a single bacterium. These adhesins are created at a constant rate ($k_{\text{on}}$) throughout the chain until reaching the maximum number of adhesins per length ($n$), and detach above a critical force ($F_{\text{link}}$). The force on a single adhesin is given by the following equation:

$$F_{\text{adh}} = -A\left(\frac{L}{L_0} + \frac{1}{4}\left(1 - \frac{L}{L_0}\right)^{-2} - \frac{1}{4}\right) \tag{13}$$

$L$ is the link extension and $L_0$ is a constant.

The detach rate of an adhesin depends on the force exerted on it. The detach rate is given by the following equation:

$$r(F_{\text{adh}}) = k_{\text{off}} \times \begin{cases} 1 + arctanh\left(\frac{F_{\text{adh}}}{F_{\text{link}}}\right), \text{if} \quad F_{\text{adh}} \leq F_{\text{link}} \\ \\ \infty, \text{ otherwise} \end{cases} \tag{14}$$

The parameters used in the simulations are listed in Supplementary Table 1.

Numeric solution of tension and buckling profiles of growing rods are given in Supplementary Figure 5. Viscous drag force provides global buckling profile. However, uniform friction and adhesive force model can provide localized tension and also trigger the localization of the buckling profiles. Supplementary Figure 5 shows the comparison of tension profiles and decay lengths of the buckling regions under different forces.

## Supplementary References

1. Duvernoy, M.-C. et al. Asymmetric adhesion of rod-shaped bacteria controls microcolony morphogenesis. *Nat Commun* **9**, 1120, doi:10.1038/s41467-018-03446-y (2018).
