# Supplementary material for: Emergence of active nematics in chaining bacterial biofilms
Source: Nat Commun. 2019 May 23;10:2285. doi: 10.1038/s41467-019-10311-z (PMC6533293; doi:10.1038/s41467-019-10311-z)
Supplement: Supplementary file 2 — Description of Additional Supplementary Files [file 41467_2019_10311_MOESM2_ESM.pdf]

**Description of Additional Supplementary Files:**

**Supplementary Video 1: Early dynamics of growing biofilm**

Starting from a single bacterium, the video shows the formation of a bacterial chain, localized buckling, and the crumpled structures. The biofilm was grown on 1.5% agar surface at 21 $^{o}$C.

**Supplementary Video 2: Emergence of active nematics**

The video shows the biofilm formation of chaining strain BAK47 on 1.5% agar surface at 21 $^{o}$C.

**Supplementary Video 3: Emergence of active nematics**

The video shows the second example of a biofilm formation on 1.5% agar surface at 21 $^{o}$C using chaining strain BAK47.

**Supplementary Video 4: The growing edge of a biofilm**

The video shows the edge of a growing biofilm. Alligned chain of cells at the edge resembles microtubule-based active nematic systems.

**Supplementary Video 5: Non-chaining bacteria**

The video shows the colony growth of non-chaining bacterial strain BAK51 on 1.5% agar surface at 21 $^{o}$C.

**Supplementary Video 6: Chaining in liquid**

The video shows the biofilm formation in liquid LB broth. Localized buckling instabilities are not observed. The chaining process was induced by IPTG based induction using strain BAK50.

**Supplementary Video 7: A growing biofilm in a liquid**

The video shows the later stage of A biofilm formation in liquid LB broth. Supercoiling process drives the formation of the biofilm in liquid.

**Supplementary Video 8: FEM simulations of edge instabilities**

The video shows the results of FEM simulation of a droplet-shaped elastic chain with a long straight tail. Color code shows the stress level.

**Supplementary Video 9: FEM simulations of multilayered circular elastic rods.**

The video shows the results of FEM simulation of multilayered circles. The initial radius is below the critical radius and simulation was performed until the secondary edge instability occurs.

**Supplementary Video 10: FEM simulations of U shaped multilayered elastic rod.**

The video shows the results of FEM simulation of multilayered U shaped structure. U shaped initial configuration give rise to more realistic colony morphology.

**Supplementary Video 11: 3D FEM simulation of an elastic rod forming vertical lift-off.**

The video shows the result of 3D FEM simulation of an elastic rod. Vertical lift-off process takes place at (-1/2) defect.
